# Wheat genetic gains for two distinct management schemes in China: An analysis of elite spring type genotypes

**DOI:** 10.1371/journal.pone.0228823

**Published:** 2020-02-06

**Authors:** Ding Mingliang, Muhammad Asim, Li Mingju, Sedhom Abdelkhalik, Daniel Manore, Li Shaoxiang, Zhao Hong, Lin Liping

**Affiliations:** 1 Institute of Food Crops, Yunnan Academy of Agricultural Sciences, Kunming, China; 2 Plant Sciences Division, Pakistan Agricultural Research Council, Islamabad, Pakistan; 3 Institute of Agricultural Environment and Resources, Yunnan Academy of Agricultural Sciences, Kunming, China; 4 Field Crops Research Institute, Agricultural Research Center, Kaferelsheikh, Egypt; 5 Department of Plant Science, College of Agriculture, Wachemo University, Hosaena, Ethiopia; 6 Seed Management Department of Yunnan Province, Kunming, China; Institute of Genetics and Developmental Biology Chinese Academy of Sciences, CHINA

## Abstract

The information collected through multi-environment testing of wheat genotypes not only provides basis to identify promising genotypes but also to ascertain their yield potential and the genetic gains. For this purpose, in the presented study, the data originated from the Yunnan provincial Regional Yield Trials (RYT) conducted during 2006 and 2018 was used. During this period, 107 genotypes were evaluated at 18 locations under Upland Wheat (UW) management scheme, while 116 genotypes were evaluated at 21 locations under Field Wheat (FW) management scheme. By adopting standard statistical approaches and through repeated elimination procedures, 7 genotypes emerged as promising for UW and 11 for FW cultivation. These genotypes have genetic variance >1 and 44/33% higher average yield than that of UW/FW genotypes. Most of these promising genotypes were tested during 2016 and 2018 cropping seasons. This indicated a good genetic gain of around 0.7 t/ha in recent years from that of base year. These genotypes, however, needs to be further evaluated in diverse environments suitable for spring type wheat cultivation to ascertain the extent of their interaction with wider environmental conditions and possibility of using in local breeding programs of those target environments.

## Introduction

As an important cereal crop, wheat is crucial in terms of global food security [1]. Owing to its enormous genetic variability in phenological response to photoperiod and temperature, it is grown in almost all regions of the world in locations ranging in altitude from a few meters to more than 3000 m above sea level [2]. Almost one sixth of the total arable land in the world is cultivated with wheat and the area under wheat cultivation is more than 240 million ha [3]. Bulk of the wheat is produced under rainfed conditions while less than 20% of the global wheat is produced under irrigated conditions [4–6].

Release of high yielding cultivars is one of the important strategies to achieve per unit area yield increments [7]. Before the release of any specific genotype for its commercial cultivation by the farmers, an important task is the multi-environment testing of this genotype for ascertaining its yield potential [8,9]. This multi-environment testing on one side provides an estimate of genotypic interaction with the testing environments [10] while on the other side the estimated G x E interaction based differential ranking of genotypes across environments may rarify the process of selecting and recommending a genotype for target environment [11].

The target environment for wheat farmers in China, which is the world leading wheat producer from around 24 million hectares, varies in the country’s spring wheat production zone (comprised of north-eastern, northern and north-western regions), winter wheat production zone (comprising northern, Huang-Huai, the middle and lower reaches of the Changjiang river, south-western and southern regions) and spring-winter wheat mixed production zone (comprised of Xinjiang and Qinghai-Tibet regions) [12].

The Yunnan Province is an important producer of spring wheat in China. Here, wheat is cultivated under two crop management schemes, namely Field Wheat (FW) and Upland Wheat (UW). FW refers to the cultivation of wheat in rice-wheat cropping pattern. Whereas, UW management refers to cultivation of wheat following maize or other dry land crop [13]. There are great differences in the characteristics and cultivation patterns of these two different types of wheat in Yunnan Province. For example, in the FW management scheme, fertilizer application is generally split in three phases, mainly at sowing, tillering and jointing. Whereas, only one fertilizer application at sowing is being practiced in UW. Moreover, crop is irrigated once in UW management scheme while it receives five irrigations in FW scheme (at seedling, tiller initiation, jointing, booting and grouting stages).

The research system comprising of institutes/academies, public/private sector as well as universities [S1 and S2 Tables] contributed in the development of new high yielding cultivars for farmers by providing elite genetic material for both of these schemes. This elite genetic material is subjected to regional level multi-environment testing like most of the global varietal release systems.

The present study was performed to 1) identify the promising genotypes for both the management schemes and 2) quantify the genetic gains over the years in breeding varieties for these schemes.

## Materials and methods

### Description of the experiments

For the purpose of this study, the data generated through Yunnan provincial Regional Yield Trials (RYT) for the duration of 2006 and 2018 was used. Each year a number of genotypes were evaluated under Upland Wheat (UW) and Field Wheat (FW) management schemes (Table 1). At each location, the experiment was conducted by adopting Randomized Complete Block Design approach in triplicate following the recommended package of production technology for each crop management scheme. Moreover, in addition to the locations each year was considered as a unique testing environment [14]. During the study period, the experiments were conducted at 18 locations under UW and 21 under FW. Out of these 7 locations were in common where both UW and FW genotypes were evaluated during the period (2006–2018) under consideration.

**Table 1:** Genotypes evaluated under RYT (2006–2018) across locations (in alphabetical order) and over years for Upland Wheat (UW) and Field Wheat (FW) management schemes.

Location	Unique testing environments		Genotypes (number)		Yield (t/ha)				Coefficient of Variation	
					Minimum		Maximum			
	UW	FW	UW	FW	UW	FW	UW	FW	UW	FW
Chuxiong	13	13	97	116	2.5	0	9.5	7.7	19.1	28.8
Dali	12	12	89	94	2.0	0	7.9	9.7	29.6	24.9
Huize	12		107		0.8		4.1		32.1	
Yimen	11		95		1.1		9.9		51.3	
Lin Xiang	9	7	85	80	1.4	0	7.5	8.3	19.9	28.3
Wenshan	9	4	85	22	2.6	2.3	7.2	6.1	20.3	16.1
Zhenxiong	9		83		0.6		6.4		22.1	
Ning ' er	8		81		1.2		6.7		28.9	
Mengzi	7		73		0		6.7		59.6	
Songming	7		73		2.9		7.8		18.9	
Qujing	5		35		1.7		7.2		26.4	
Lincang	4	6	22	37	3.3	4.5	7.2	7.7	17.1	12.7
Zhaotong	4		35		1.3		5.5		32.1	
Kunming	3	5	24	37	2.8	3.0	6.3	7.9	21.1	21.7
Honghe	3	2	19	10	2	3.0	4.9	5.7	25.9	18.8
Pu’er	2		13		4.2		5.4		6.0	
Simao	2		10		4.2		6.3		11.8	
Nanjian	1		21		3.9		7.8		18.1	
Hongta		7		80		0		9		38.4
Jingdong		7		80		0		8.4		28.4
Longyang		7		80		0		8.5		26.9
Midu		7		61		1.8		11.3		32.3
Yiliang		7		80		0		10.3		36.2
Yulong		7		80		0		10.5		33.7
Baoshan		6		37		1.7		7.8		25.9
Dehong		6		37		5.0		8.9		12.2
Lijiang		6		37		3.8		11.1		29
Luxi		6		58		0		9.5		29.9
Yuxi		6		37		2.3		8.7		20.9
Zhaoyang		6		58		0		6.2		47.8
Maitreya		4		28		3.2		7.2		18.1
Mangshi		1		24		5.4		8.7		14.4

### The study material and data

RYT for UW and FW were comprised of elite genetic material supplied by different provincial research establishments [S1 and S2 Tables]. The data generated for genotypes tested under both the management schemes is summarized in Table 2 as averaged across locations and in Tables 3 as averaged across years. The mean grain yield values of these genotypes were used for this study and subjected to further analysis.

**Table 2:** Location wise performance statistics of Upland Wheat (UW) and Field Wheat (FW) genotypes.

Location	Trial Mean Yield (t/ha)		Standard Error		Dispersion of observations (t/ha)		Variability of observations	
	UW	FW	UW	FW	UW	FW	UW	FW
Nanjian	5.82		0.23		1.06		1.11	
Chuxiong	5.61	5.02	0.08	0.10	1.07	1.45	1.15	2.10
Lincang	5.60	6.14	0.14	0.09	0.96	0.78	0.91	0.61
Lin Xiang	5.58	5.82	0.09	0.15	1.11	1.65	1.23	2.71
Songming	5.27		0.09		1.00		1.00	
Simao	5.10		0.13		0.60		0.36	
Wenshan	5.03	4.40	0.09	0.10	1.02	0.71	1.04	0.51
Qujing	4.70		0.16		1.24		1.54	
Pu’er	4.50		0.05		0.27		0.07	
Zhenxiong	4.48		0.09		0.99		0.98	
Kunming	4.36	5.71	0.16	0.18	0.92	1.24	0.85	1.54
Ning ' er	4.17		0.11		1.20		1.45	
Dali	4.16	6.40	0.10	0.12	1.23	1.59	1.51	2.54
Yimen	4.15		0.17		2.13		4.54	
Zhaotong	3.52		0.16		1.13		1.28	
Honghe	3.02	4.24	0.14	0.18	0.78	0.80	0.61	0.64
Mengzi	2.56		0.14		1.53		2.33	
Huize	2.23		0.05		0.72		0.51	
Mangshi		7.28		0.21		1.05		1.10
Dehong		6.98		0.10		0.85		0.72
Luxi		6.76		0.20		2.02		4.09
Midu		6.47		0.21		2.09		4.38
Yulong		6.31		0.19		2.12		4.51
Lijiang		6.11		0.21		1.77		3.14
Longyang		6.08		0.14		1.64		2.68
Hongta		5.98		0.20		2.30		5.27
Yiliang		5.77		0.18		2.09		4.36
Yuxi		5.59		0.14		1.17		1.37
Jingdong		5.41		0.14		1.53		2.35
Maitreya		5.20		0.15		0.94		0.89
Baoshan		4.93		0.15		1.27		1.62
Zhaoyang		3.53		0.17		1.69		2.85

**Table 3:** Performance statistics of Upland Wheat (UW) and Field Wheat (FW) genotypes within each unique testing environment.

Year	Number of tested				Trial Mean Yield (t/ha)		Standard Error		Dispersion of observations (t/ha)		Variability of observations	
	Genotypes		Locations									
	UW	FW	UW	FW	UW	FW	UW	FW	UW	FW	UW	FW
2006	10	10	10	12	4.16	6.36	0.13	0.14	1.34	1.48	1.79	2.19
2007	10	10	11	12	3.86	5.87	0.13	0.12	1.36	1.22	1.84	1.49
2008	13	13	6	12	5.02	5.2	0.11	0.13	0.91	1.46	0.83	2.13
2009	13	13	7	11	3.94	5.46	0.11	0.09	1.09	1.08	1.19	1.16
2010	15	15	9	11	3.51	5.27	0.14	0.11	1.58	1.38	2.51	1.91
2011	11	15	9	11	4.63	5.89	0.15	0.16	1.46	1.48	2.13	2.19
2012	15	15	10	11	4.02	5.35	0.14	0.19	1.67	2.34	2.8	5.48
2013	11	15	10	11	3.51	4.52	0.14	0.26	1.44	3.13	2.07	9.78
2014	18	19	10	11	4.29	5.88	0.11	0.09	1.57	1.21	2.46	1.46
2015	15	19	10	11	4.76	5.39	0.15	0.17	1.89	2.33	3.58	5.44
2016	19	21	10	11	4.96	6.11	0.11	0.09	1.6	1.25	2.56	1.57
2017	12	14	10	11	4.53	5.74	0.16	0.12	1.74	1.39	3.02	1.93
2018	21	25	10	10	4.81	7.16	0.12	0.09	1.63	1.29	2.67	1.67

### The statistical analysis

The grain yield assessed for each genotype at a particular location and under each unique testing environment was subjected to a series of statistical analyses [15](Table 4). The parameters of stability statistics [16–18] were also obtained for these genotypes.

**Table 4:** Summarized statistical output for yield data of Upland Wheat (UW) and Field Wheat (FW)-RYT 2006–2018.

Analysis of Variance											Yield (t/ha)	
Source	DF		MS		Pr> F		CV		RMSE			
	UW	FW	UW	FW	UW	FW	UW	FW	UW	FW	UW	FW
*GLM One way*											4.35	5.76
Model (Genotypes, G)	106	115	3.61	19.11	0.0059	<0.0001	37	27	1.6	1.6		
Model (Locations, L)	17	20	119	64.57	<0.0001	<0.0001	28	29	1.2	1.7		
Model (Unique testing environment, Y)	12	12	34.3	73.54	<0.0001	<0.0001	36	30	1.6	1.7		
*GLM N-Way*												
L	17	20	120.8	61.4	<0.0001	<0.0001	12	7	0.5	0.4		
Y	12	10	34.32	14	<0.0001	<0.0001						
G	106	113	1.26	12.8	<0.0001	<0.0001						
Y x L	91	78	14.05	8	<0.0001	<0.0001						
G x Y	64	69	0.34	10.7	0.1947	<0.0001						
G x L	914	1018	0.56	0.8	0.0052	<0.0001						
G x Y x L	475	606	0.24	0.4	0.6619	0.0002						

In order to identify the promising genotypes, we adopted a simple approach wherein mean genetic deviation was calculated successively. To start with, it was calculated from 107 UW genotypes and 116 FW genotypes using the expression

$$Y_{i}=Y_{j,k}-{\bar{Y}}_{i,j,k}$$

which gives the mean genetic deviation of *i*^*th*^ genotype from its deviation at *j*^*th*^ year and *k*^*th*^ location. The repeated use of this expression with genotypes having corresponding above mean values facilitate the partitioning of these genotypes, location and years to identify the promising genotypes and genetic gain during this duration.

## Results and discussion

### Genotypic performance

The trial mean yield of upland wheat (UW) genotypes varied between 2–6 t/ha whereas that of field wheat (FW) genotypes varied between 3–7 t/ha across locations (Table 2). Over the years, these genotypes exhibited a range of 3–5 t/ha and 4–7 t/ha for UW and FW genotypes respectively (Table 3). This might be attributed to higher yielding abilities of FW genotypes [13]. Overall, the variations in UW genotypes was 36% more than that of FW genotypes. Within the group variations over years was more for UW genotypes whereas FW genotypes exhibited slightly higher variations over locations. These differences were significant and exhibited a great diversity among these genotypes as well as their performance across locations and over the years (Table 4).

**Fig 1:**
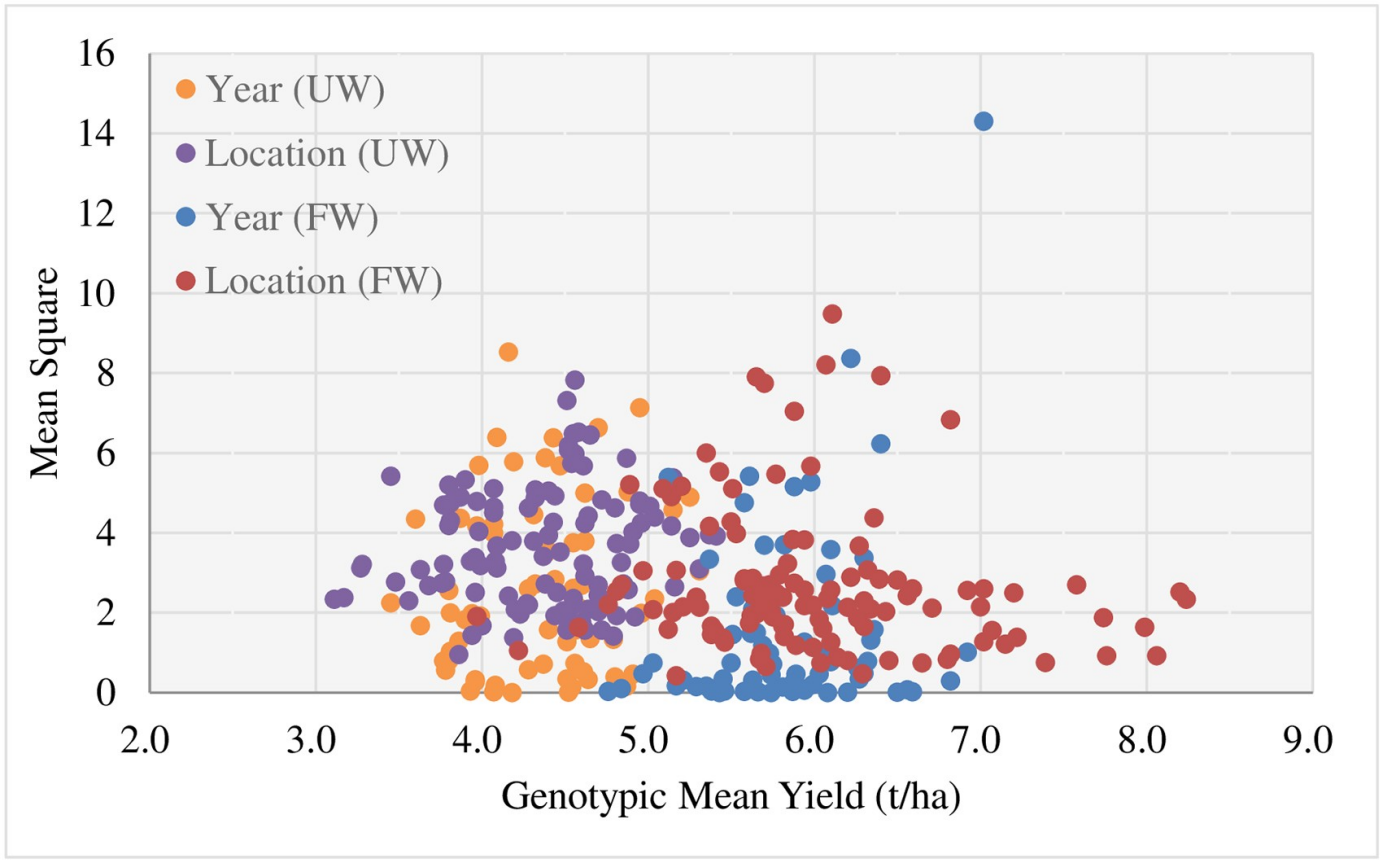
Genotypic performance over years and across locations calculated from individual trial analysis.

Considerable variations in trial mean square values and trial mean yield was observed. The highest mean square values over years for FW genotypes were of greater extent (~68%) than that of UW genotypes. This was considerably low (11%) for locations (Fig 1).

This variable performance over years and locations for both UW and FW genotypes indicated that the results lacking any predictable order. This might be due to varying number of genotypes tested over locations and years. This noise in results necessitated the consistency in testing environments and locations to achieve the selection gains mediated through such trials by minimizing the influence of such variations [19].

### Genotypic selection

A number of approaches have been reported based on various multilocational studies to facilitate genotypic selection considering the genetic and environmental factors [8, 9, 20, 21]. In this study, the trial data was exploited to extract as much information as possible through a simple and easy to apply approach for ascertain the promising genotypes.

**Fig 2:**
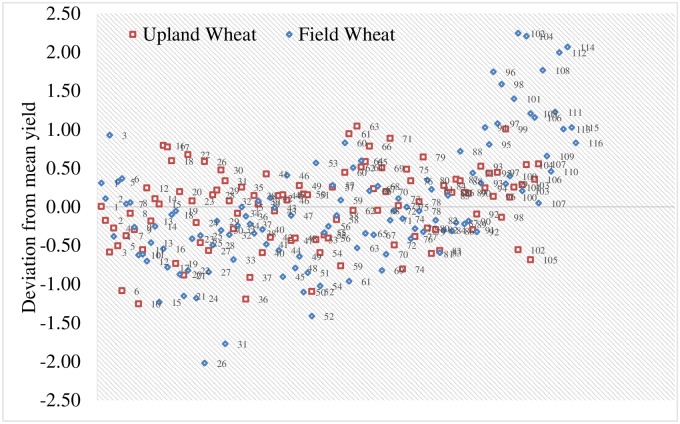
Mean genetic deviations obtained in the first cycle.

**Fig 3:**
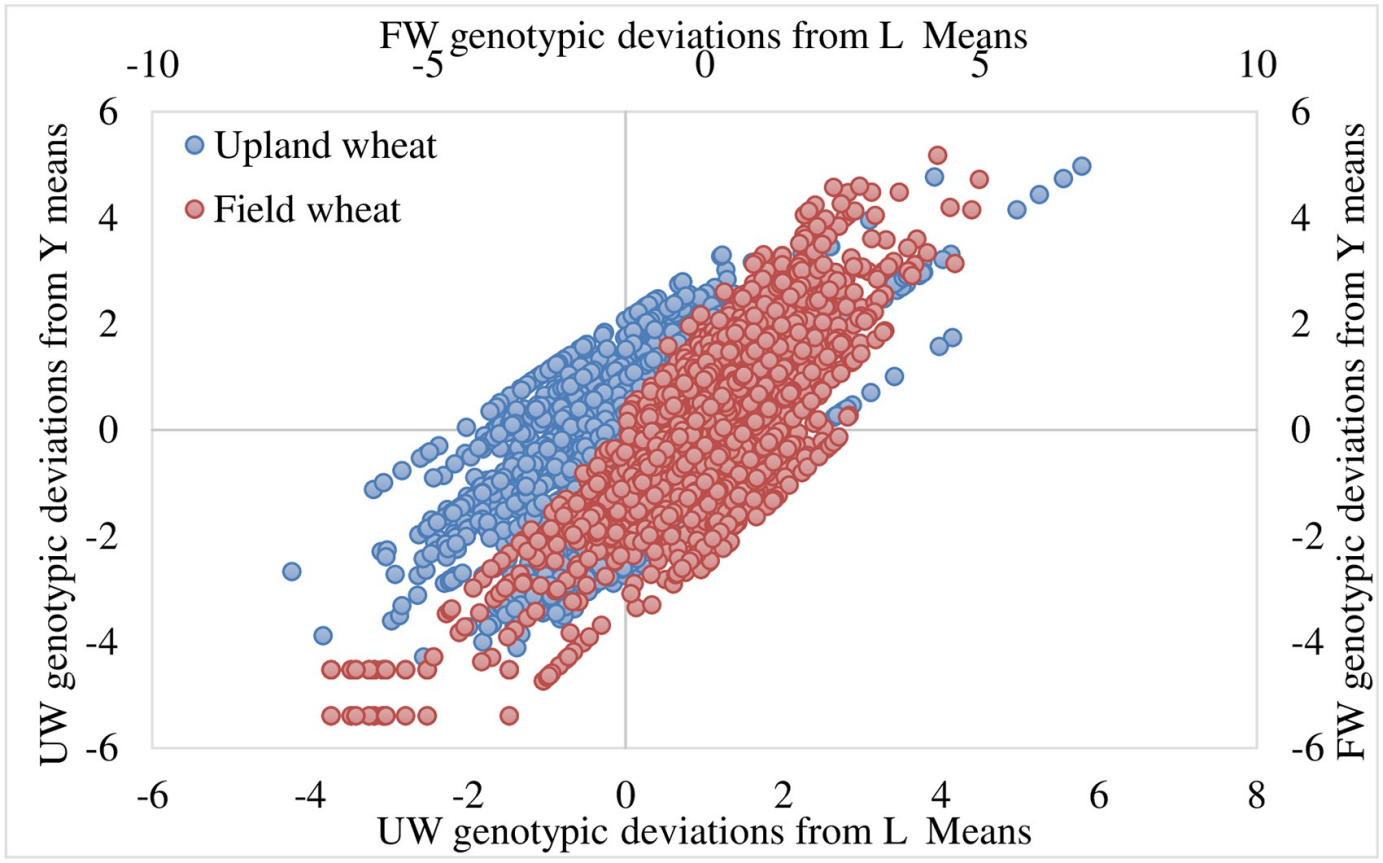
Mean genetic deviations obtained in the second cycle.

**Fig 4:**
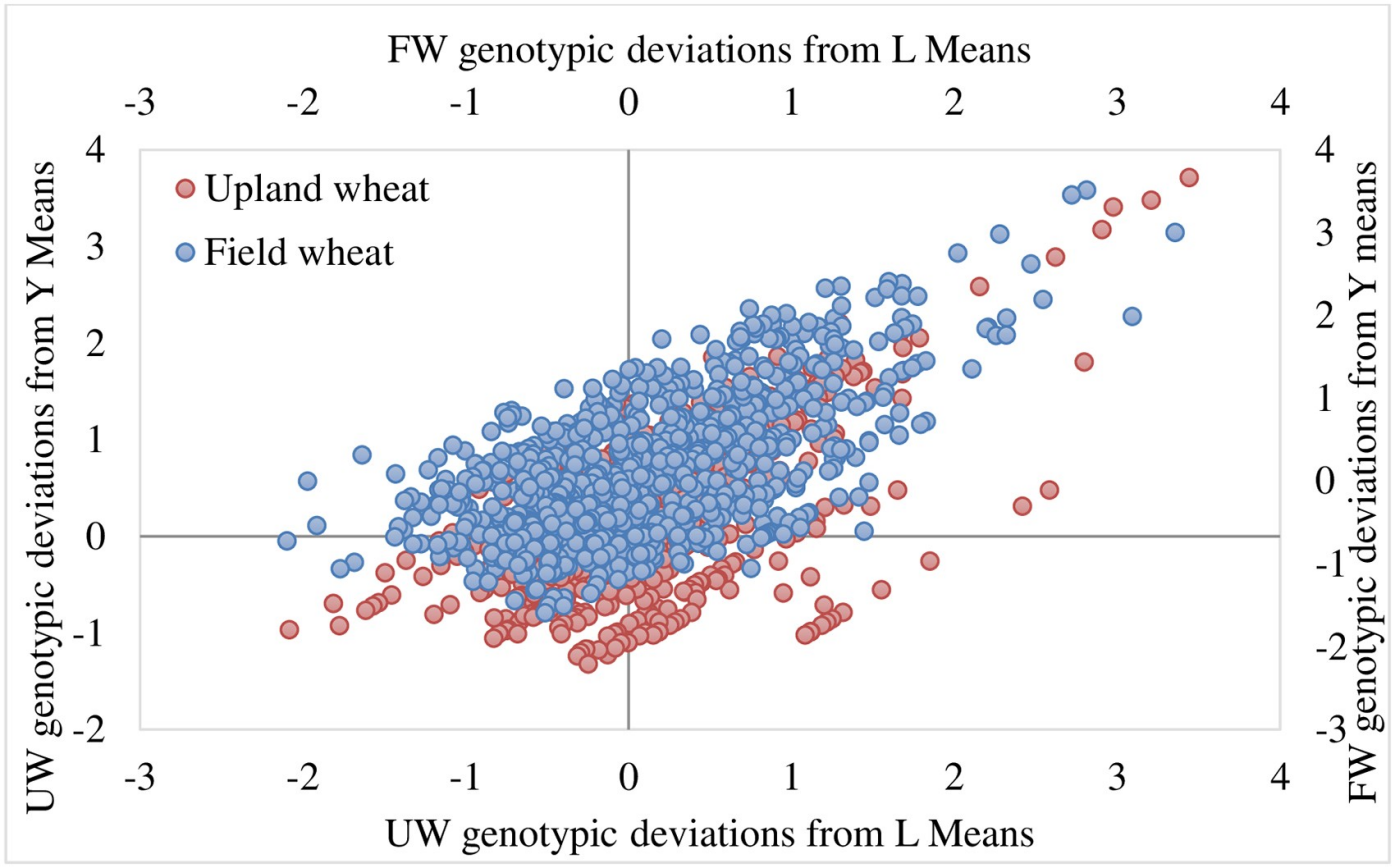
Mean genetic deviations obtained in the third cycle.

Through a repeated elimination process of sub-optimal performing genotypes, 57% of the UW genotypes and 43% of the FW genotypes were excluded in the first cycle (Fig 2). During the subsequent cycles (Figs 3 and 4), genotypes were eliminated considering the prevailing environmental factors (years and locations) [21].

**Fig 5:**
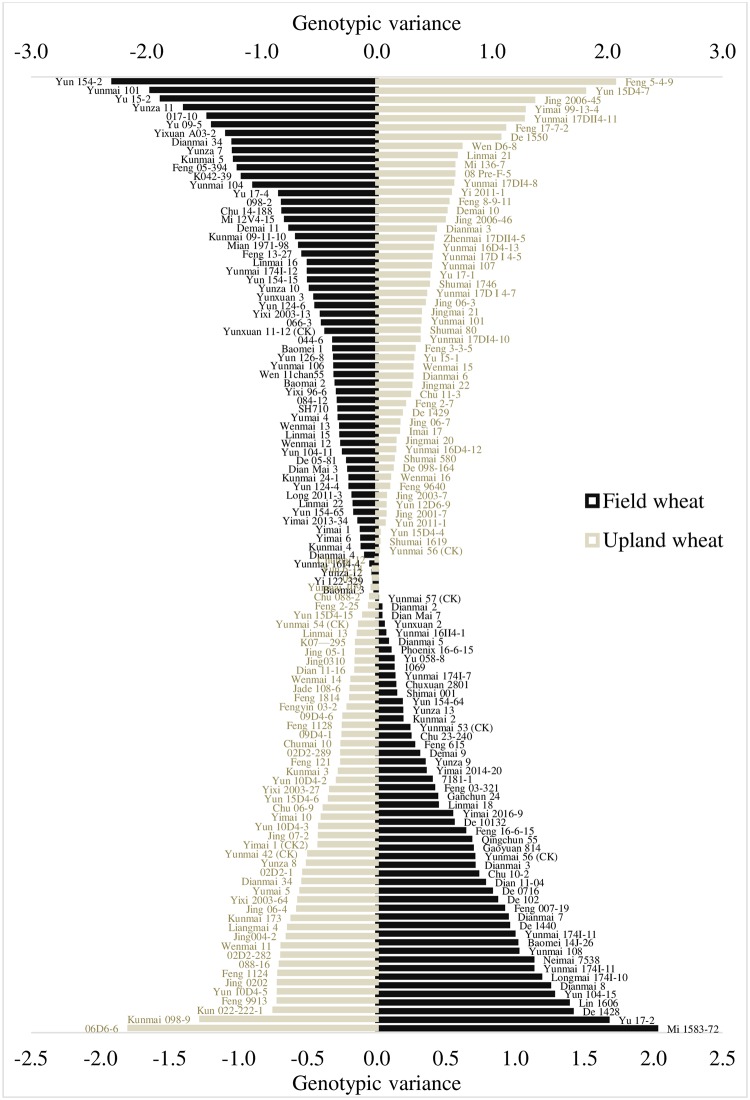
Promising genotypes for upland and field wheat cultivation.

**Fig 6:**
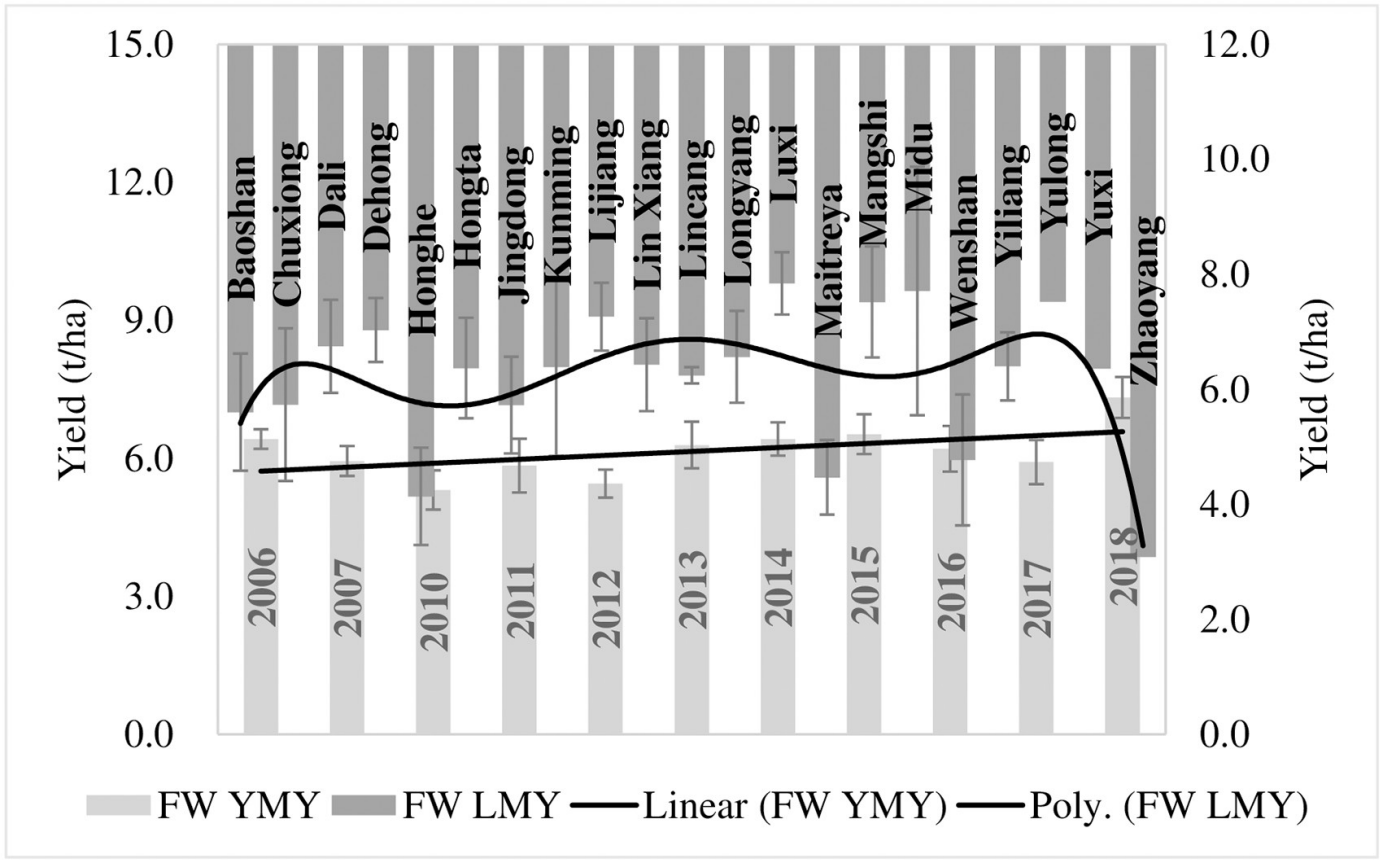
A summed-up performance of selected FW genotypes over locations and years.

**Fig 7:**
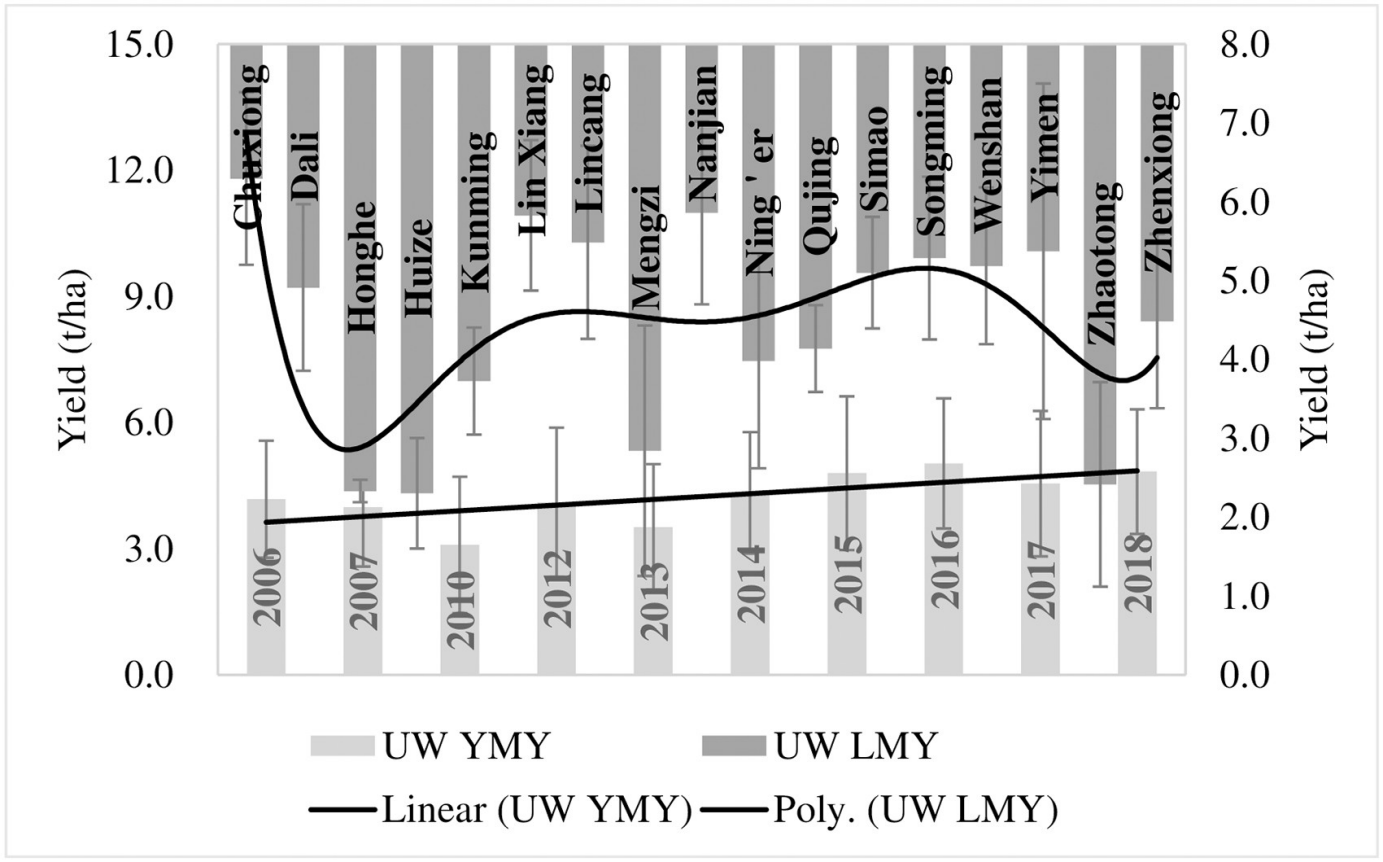
A summed-up performance of selected UW genotypes over locations and years.

Resultantly, the genotypes [Feng 5-4-9, Yun 15D4-7, Jing 2006–45, Yimai 99-13-4 and Feng 17-7-2 (tested during 2016), Yunmai 17DII4-11 and De 1550 (tested during 2018)] emerged as promising for upland wheat management scheme. These genotypes have genetic variance > 1 and their averaged yield was 44% higher than the average of yield of all UW genotypes. Similarly, for field wheat management scheme, genotypes Mi 1583–72, Yu 17–2, De 1428, Lin 1606, Dianmai 8, Longmai 174I-10, Yunmai 174I-11, Neimai 7538, Yunmai 108 and Baomei 14J-26 (tested during 2018) and Yun 104–15 (tested during 2012–13) emerged as promising by having >1 genetic variance and average yield higher by 33% from the average yield of all FW genotypes (Figs 5–7).

### Genetic gains

The partitioning of selected genotypes over locations and years indicated yield variations of around 4 tons across locations and 2 tons across years for UW genotypes. This variation was 16% higher for FW genotypes across locations. Whereas, it was around 4% higher across years for FW genotypes.

These differences in genetic gains between FW and UW were mainly attributed to the crop management strategies in these schemes. In UW scheme, wheat was sown after the harvest of dry land crops such as corn whereas, FW represented the post rice wheat. In addition to these management differences, other possible causes of observed variations of wheat in UW scheme could be the type which is recognized as a weak spring or semi winter wheat variety with drought resistance, barren resistance, cold resistance and strong tillering ability. On the other hand, FW is mostly spring variety with high and stable yield but weak tillering ability in the studied ecologies.

**Fig 8:**
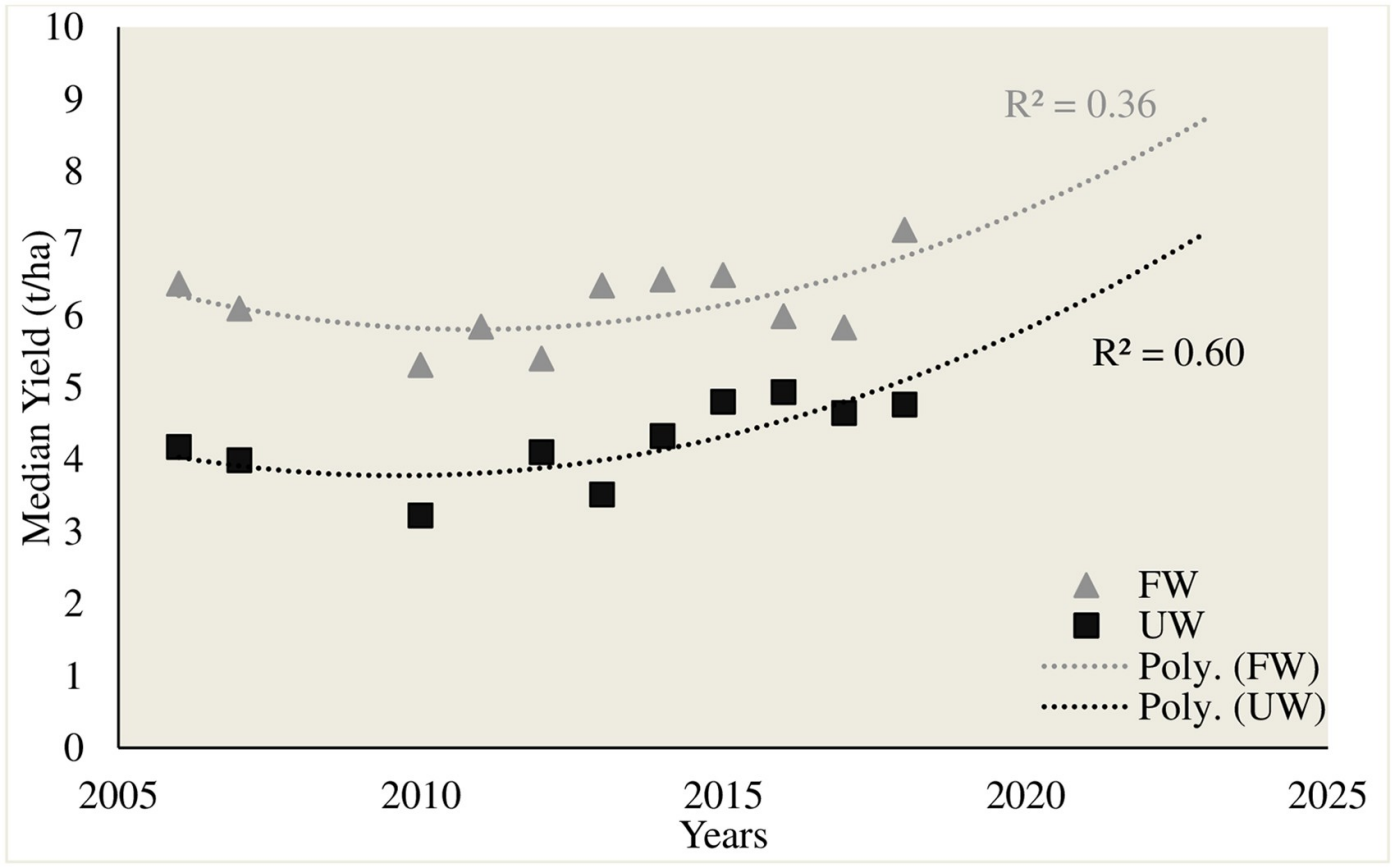
Genetic gains in field and upland wheat breeding programs.

The data also indicated that interestingly, the promising UW genotypes were tested during 2016 and 2018 cropping seasons. Whereas, most of the promising FW genotypes were tested during 2018 cropping season. This observed shrinkage of promising genotypes in recent years indicated a good genetic gain in later part of this study. Given that, the rate of genetic gain in wheat has declined over the past decade [22], this progress appeared encouraging to tackle the growing demand of wheat for the projected 9 billion population by 2050 [23]. Quantification of this, exhibited a genetic gain of around 0.7 t/ha in FW genotypes and 0.6 t/ha in UW genotypes from the base year in this study (Fig 8). The genetic potential of these promising genotypes needs to be evaluated in other environments having spring type bread wheat under cultivation. Moreover, these could also be utilized in breeding programs for those target environments to enhance the genetic diversity and increase yield potential.

## Conclusions

The varied performance of both UW and FW genotypes, over years and locations, indicated the lack of any predictable order. Summarizing these results suggested more variations in UW genotypes as compared to that of FW genotypes. Given that, it is recommended to evaluate these in wider environmental conditions and utilize in breeding programs for enhancing genetic diversity and genetic gains.

## Supporting information

S1 Table Details about the upland wheat genotypes and their performance over years and locations. (DOCX)

S2 Table Details about the field wheat genotypes and their performance over years and locations. (DOCX)
