# Supplementary material for: Wheat genetic gains for two distinct management schemes in China: An analysis of elite spring type genotypes
Source: PLoS One. 2020 Feb 6;15(2):e0228823. doi: 10.1371/journal.pone.0228823 (PMC7004340; doi:10.1371/journal.pone.0228823)
Supplement: S1 Table — (DOCX) [file pone.0228823.s001.docx]

**S1 Table.**

| **Genotype** | **Breeding Program** | **Year(s) of testing** | **Mean Yield (t/ha)** | **Range (t/ha)** |
| --- | --- | --- | --- | --- |
| 02D2-1 | The Institute of Food Crops (IFC),Yunnan Academy of Agriculture Sciences (YAAS) | 2006, 2007 | 4.37 | 4.12 |
| 02D2-282 | IFC, YAAS | 2008, 2009 | 4.19 | 4.41 |
| 02D2-289 | IFC, YAAS | 2006, 2007 | 3.78 | 4.17 |
| 05-1 | Wenshan Academy of Agricultural Sciences (WAAS) | 2010-2011 | 4.09 | 5.43 |
| 06D6-6 | IFC, YAAS | 2008, 2009 | 3.86 | 3.05 |
| 08 Pre-F-5 | IFC, YAAS | 2011 | 3.28 | 5.45 |
| 088-16 | Agricultural Technology Extension Center of Mile (ATECM) | 2010, 2011 | 3.99 | 4.82 |
| 09D4-1 | IFC, YAAS | 2010, 2011 | 4.28 | 5.59 |
| 09D4-6 | IFC, YAAS | 2010, 2011 | 4.07 | 5.14 |
| Chu 06-9 | Chuxiong Academy of Agricultural Sciences (ChAAS) | 2010 | 3.11 | 4.88 |
| Chu 088-2 | ChAAS | 2012, 2013 | 3.81 | 5.08 |
| Chu 11-3 | ChAAS | 2014, 2015 | 4.61 | 7.28 |
| Chumai 10 | ChAAS | 2006, 2007 | 4.18 | 5.38 |
| Chumai 12 | ChAAS | 2008, 2009 | 4.47 | 5.57 |
| De 098-164 | Dehong Institute of Agricultural Sciences (DIAS) | 2014, 2015 | 4.40 | 5.68 |
| De 1429 | DIAS | 2018 | 5.16 | 4.64 |
| De 1550 | DIAS | 2018 | 5.14 | 5.54 |
| Demai 10 | DIAS | 2016, 2017 | 4.96 | 6.60 |
| Dian 11-16 | Yunnan Agricultural University (YAU) | 2012, 2013 | 3.63 | 4.24 |
| Dianmai 3 | YAU | 2014, 2015 | 4.56 | 8.25 |
| Dianmai 34 | YAU | 2012 | 3.48 | 4.30 |
| Dianmai 6 | YAU | 2016, 2017 | 5.04 | 7.41 |
| Feng 1124 | Dali Academy of Agricultural Sciences (DAAS) | 2008, 2009 | 4.44 | 3.75 |
| Feng 1128 | DAAS | 2010, 2011 | 4.16 | 5.29 |
| Feng 121 | DAAS | 2012, 2013 | 3.90 | 5.96 |
| Feng 17-7-2 | DAAS | 2016 | 4.95 | 7.85 |
| Feng 1814 | DAAS | 2012, 2013 | 3.80 | 5.74 |
| Feng 2-25 | DAAS | 2014, 2015 | 4.52 | 6.72 |
| Feng 2-7 | DAAS | 2014, 2015 | 4.58 | 7.05 |
| Feng 3-3-5 | DAAS | 2018 | 4.84 | 5.03 |
| Feng 5-4-9 | DAAS | 2016 | 4.70 | 5.86 |
| Feng 8-9-11 | DAAS | 2018 | 4.44 | 5.88 |
| Feng 9640 | DAAS | 2006, 2007 | 4.08 | 5.00 |
| Feng 9913 | DAAS | 2006, 2007 | 4.28 | 3.70 |
| Fengyin 03-2 | DAAS | 2008, 2009 | 4.62 | 4.59 |
| Imai 17 | Lincang Institute of Agricultural Sciences (LIAS) | 2010 | 3.17 | 4.95 |
| Jade 108-6 | Yuxi Academy of Agricultural Sciences (YuAAS) | 2012, 2013 | 3.45 | 5.83 |
| Jing 0202 | Agricultural Technology Extension Center of Qujing (ATECQ) | 2008, 2009 | 4.51 | 3.36 |
| Jing 05-1 | ATECQ | 2010, 2011 | 4.40 | 5.68 |
| Jing 06-3 | ATECQ | 2012 | 3.77 | 5.21 |
| Jing 06-4 | ATECQ | 2008, 2009 | 4.79 | 3.63 |
| Jing 06-7 | ATECQ | 2012, 2013 | 3.97 | 6.11 |
| Jing 07-2 | ATECQ | 2010, 2011 | 4.31 | 4.69 |
| Jing 2001-7 | Qujing Academy of Agricultural Sciences (QAAS) | 2014, 2015 | 4.51 | 7.23 |
| Jing 2003-7 | QAAS | 2014, 2015 | 4.52 | 6.71 |
| Jing 2006-45 | QAAS | 2016 | 4.45 | 5.59 |
| Jing 2006-46 | QAAS | 2016 | 4.92 | 5.21 |
| Jing004-2 | QAAS | 2006, 2007 | 3.93 | 4.56 |
| Jing0310 | QAAS | 2006, 2007 | 3.96 | 4.78 |
| Jingmai 20 | QAAS | 2018 | 4.64 | 4.77 |
| Jingmai 21 | QAAS | 2018 | 4.53 | 4.31 |
| Jingmai 22 | QAAS | 2018 | 4.52 | 4.26 |
| K07—295 | Kunming Academy of Agricultural Sciences (KAAS) | 2010 | 3.27 | 4.84 |
| Kun 022-222-1 | KAAS | 2008, 2009 | 3.94 | 3.47 |
| Kunmai 098-9 | KAAS | 2012, 2013 | 3.77 | 4.85 |
| Kunmai 173 | KAAS | 2014 | 4.00 | 4.22 |
| Kunmai 3 | KAAS | 2006, 2007 | 3.96 | 4.14 |
| Liangmai 4 | Sichuan Agricultural University (SAU) | 2008, 2009 | 4.62 | 4.00 |
| Lin 1610 | LIAS | 2018 | 4.20 | 4.20 |
| Linmai 13 | LIAS | 2006, 2007 | 3.60 | 4.89 |
| Linmai 21 | LIAS | 2016 | 4.81 | 6.74 |
| Mi 136-7 | ATECM | 2016, 2017 | 5.31 | 8.02 |
| Shumai 1619 | YAU | 2017 | 4.32 | 5.69 |
| Shumai 1746 | YAU | 2018 | 5.41 | 4.63 |
| Shumai 580 | YAU | 2014, 2015 | 4.88 | 6.91 |
| Shumai 80 | YAU | 2016, 2017 | 4.95 | 6.16 |
| Wen D6-8 | WAAS | 2016, 2017 | 5.15 | 8.17 |
| Wenmai 11 | WAAS | 2008, 2009 | 4.60 | 3.83 |
| Wenmai 14 | WAAS | 2012, 2013 | 4.32 | 5.05 |
| Wenmai 15 | WAAS | 2014, 2015 | 4.87 | 7.14 |
| Wenmai 16 | WAAS | 2014, 2015 | 4.56 | 7.64 |
| Yi 2011-1 | Agricultural Technology Extension Center of Yimen (ATECYm) | 2016, 2017 | 5.25 | 8.07 |
| Yimai 1 (CK2) | Seed Management Department of Yiliang (SMDY) | 2010, 2011, 2012, 2013 | 3.87 | 5.33 |
| Yimai 10 | Agricultural Technology Extension Center of Yiliang (ATECYl) | 2008, 2009 | 4.38 | 4.91 |
| Yimai 96-6 | SMDY | 2014 | 3.56 | 4.25 |
| Yimai 99-13-4 | SMDY | 2016 | 4.85 | 6.13 |
| Yixi 2003-27 | SMDY | 2008, 2009 | 4.70 | 4.27 |
| Yixi 2003-64 | SMDY | 2010, 2011 | 3.98 | 5.06 |
| Yu 15-1 | YuAAS | 2016, 2017 | 4.43 | 7.01 |
| Yu 17-1 | YuAAS | 2018 | 5.01 | 6.15 |
| Yumai 5 | YuAAS | 2014 | 4.09 | 4.92 |
| Yun 10D4-2 | IFC, YAAS | 2012 | 3.76 | 4.91 |
| Yun 10D4-3 | IFC, YAAS | 2012, 2013 | 4.07 | 4.84 |
| Yun 10D4-5 | IFC, YAAS | 2012, 2013 | 3.80 | 4.59 |
| Yun 12D6-9 | IFC, YAAS | 2014, 2015 | 4.64 | 5.02 |
| Yun 15D4-15 | IFC, YAAS | 2016, 2017 | 4.55 | 6.07 |
| Yun 15D4-4 | IFC, YAAS | 2016 | 4.55 | 4.47 |
| Yun 15D4-6 | IFC, YAAS | 2016 | 4.72 | 3.59 |
| Yun 15D4-7 | IFC, YAAS | 2016 | 4.70 | 5.47 |
| Yun 2011-1 | IFC, YAAS | 2014, 2015 | 4.55 | 6.54 |
| Yun 6-14 | IFC, YAAS | 2014, 2015 | 4.54 | 6.10 |
| Yunmai 101 | IFC, YAAS | 2014, 2015 | 4.07 | 6.38 |
| Yunmai 107 | IFC, YAAS | 2016 | 4.27 | 5.13 |
| Yunmai 109 | IFC, YAAS | 2018 | 4.89 | 5.64 |
| Yunmai 16D4-12 | IFC, YAAS | 2017 | 4.61 | 5.53 |
| Yunmai 16D4-13 | IFC, YAAS | 2017, 2018 | 4.80 | 6.10 |
| Yunmai 17D I 4-5 | IFC, YAAS | 2018 | 4.50 | 4.56 |
| Yunmai 17D I 4-7 | IFC, YAAS | 2018 | 4.81 | 4.38 |
| Yunmai 17DI4-10 | IFC, YAAS | 2018 | 4.23 | 4.11 |
| Yunmai 17DI4-8 | ATECYm | 2018 | 5.37 | 4.73 |
| Yunmai 17DII4-10 | IFC, YAAS | 2018 | 4.49 | 4.54 |
| Yunmai 17DII4-11 | IFC, YAAS | 2018 | 4.62 | 5.64 |
| Yunmai 42 (CK) | IFC, YAAS | 2006, 2007, 2008, 2009, 2010, 2011 | 3.81 | 5.17 |
| Yunmai 54 (CK) | IFC, YAAS | 2010, 2011, 2014, 2015, 2016 | 4.65 | 6.16 |
| Yunmai 56 (CK) | IFC, YAAS | 2017, 2018 | 4.91 | 5.70 |
| Yunza 8 | IFC, YAAS | 2012 | 3.68 | 4.42 |
| Zhenmai 17DII4-5 | Agricultural Technology Extension Center of Zhenxiong (ATECZ) | 2018 | 4.72 | 5.69 |
