# Supplementary material for: Wheat genetic gains for two distinct management schemes in China: An analysis of elite spring type genotypes
Source: PLoS One. 2020 Feb 6;15(2):e0228823. doi: 10.1371/journal.pone.0228823 (PMC7004340; doi:10.1371/journal.pone.0228823)
Supplement: S2 Table — (DOCX) [file pone.0228823.s002.docx]

**S2 Table.**

| **Genotype** | **Breeding Program** | **Year(s) of testing** | **Mean Yield (t/ha)** | **Range (t/ha)** |
| --- | --- | --- | --- | --- |
| 1069 | IFC, YAAS | 2006, 2007 | 6.03 | 5.55 |
| 017-10 | IFC, YAAS | 2008, 2009 | 4.84 | 5.52 |
| 044-6 | IFC, YAAS | 2006, 2007 | 6.05 | 4.03 |
| 066-3 | IFC, YAAS | 2010, 2011 | 5.99 | 5.12 |
| 084-12 | IFC, YAAS | 2010, 2011 | 5.87 | 5.69 |
| 098-2 | Baoshan Institute of Agricultural Sciences (BIAS) | 2010, 2011 | 5.69 | 4.81 |
| 7181-1 | YuAAS | 2006, 2007 | 6.92 | 5.98 |
| Baomai 2 | BIAS | 2016 | 5.85 | 6.02 |
| Baomai 3 | BIAS | 2016, 2017 | 5.82 | 5.36 |
| Baomei 1 | BIAS | 2014, 2015 | 5.74 | 3.99 |
| Baomei 14J-26 | BIAS | 2018 | 6.82 | 2.93 |
| Chu 10-2 | ChAAS | 2012, 2013 | 5.88 | 8.31 |
| Chu 14-188 | ChAAS | 2016 | 5.71 | 2.46 |
| Chu 2008Jian-4 | ChAAS | 2010 | 4.22 | 3.32 |
| Chu 23-240 | ChAAS | 2006, 2007 | 6.32 | 6.28 |
| Chuxuan 2801 | ChAAS | 2014, 2015 | 6.56 | 4.21 |
| De 05-81 | DIAS | 2008, 2009 | 5.53 | 5.82 |
| De 0716 | DIAS | 2010, 2011 | 5.49 | 5.74 |
| De 10132 | DIAS | 2014, 2015 | 6.59 | 4.62 |
| De 102 | DIAS | 2012, 2013 | 6.11 | 8.32 |
| De 1428 | DIAS | 2018 | 8.21 | 5.44 |
| De 1440 | DIAS | 2018 | 7.22 | 3.95 |
| Demai 11 | DIAS | 2016 | 5.41 | 4.07 |
| Demai 9 | DIAS | 2016 | 5.99 | 3.82 |
| Dian 11-04 | YAU | 2012, 2013 | 6.08 | 7.93 |
| Dian Mai 3 | YAU | 2017 | 5.81 | 3.23 |
| Dian Mai 7 | YAU | 2017 | 6.43 | 3.96 |
| Dianmai 2 | YAU | 2014, 2015 | 6.27 | 4.16 |
| Dianmai 3 | YAU | 2018 | 6.8 | 3.38 |
| Dianmai 34 | YAU | 2010 | 4.81 | 3.92 |
| Dianmai 4 | YAU | 2016, 2017 | 6.1 | 5.12 |
| Dianmai 5 | YAU | 2016, 2017 | 6.22 | 5.88 |
| Dianmai 7 | YAU | 2018 | 7.74 | 3.99 |
| Dianmai 8 | YAU | 2018 | 7.07 | 4.28 |
| Feng 007-19 | DAAS | 2012, 2013 | 6.4 | 9.08 |
| Feng 03-321 | DAAS | 2006, 2007 | 6.36 | 7.52 |
| Feng 05-394 | DAAS | 2008, 2009 | 5.29 | 5.26 |
| Feng 13-27 | DAAS | 2014, 2015 | 4.97 | 5.06 |
| Feng 16-6-15 | DAAS | 2016 | 6.71 | 4.92 |
| Feng 615 | DAAS | 2010, 2011 | 5.63 | 5.53 |
| Ganchun 24 | YAU | 2012, 2013 | 5.65 | 8.5 |
| Gaoyuan 814 | Chinese Academy of Sciences (CAS) | 2018 | 7.02 | 3.14 |
| HX-06-1 | Ningxia Jinling Crop Research Institute (NJCRI) | 2010 | 3.97 | 4.28 |
| K042-39 | KAAS | 2010 | 5.31 | 5.24 |
| Kunmai 09-11-10 | KAAS | 2012, 2013 | 5.51 | 7.1 |
| Kunmai 2 | KAAS | 2006, 2007 | 5.61 | 5.28 |
| Kunmai 24-1 | KAAS | 2014, 2015 | 5.66 | 5.26 |
| Kunmai 4 | KAAS | 2008, 2009 | 5.74 | 4.42 |
| Kunmai 5 | KAAS | 2008, 2009 | 4.76 | 3.94 |
| Lin 1606 | LIAS | 2018 | 7.58 | 5.84 |
| Linmai 15 | LIAS | 2008, 2009 | 5.94 | 4.42 |
| Linmai 16 | LIAS | 2010, 2011 | 5.81 | 3.7 |
| Linmai 18 | LIAS | 2012, 2013 | 5.98 | 7.73 |
| Linmai 22 | LIAS | 2016 | 6.14 | 3.39 |
| Long 2011-3 | Agricultural Technology Extension Center of Longyang (ATECLy) | 2014, 2015 | 5.88 | 4.14 |
| Longmai 174I-10 | ATECLy | 2018 | 7 | 4.34 |
| Mi 12V4-15 | ATECM | 2014, 2015 | 5.46 | 4.02 |
| Mi 1583-72 | ATECM | 2018 | 7.99 | 3.34 |
| Mian 1971-98 | Mianyang Institute of Agricultural Sciences (MIAS) | 2008, 2009 | 5.12 | 4.2 |
| Neimai 7538 | ChAAS | 2018 | 8.06 | 2.34 |
| Phoenix 16-6-15 | DAAS | 2017 | 5.77 | 4.54 |
| Qingchun 55 | Lijiang Institute of Agricultural Sciences (LjIAS) | 2006, 2007 | 5.73 | 6.13 |
| SH710 | Sichuan Academy of Agricultural Sciences (SAAS) | 2010, 2011 | 5.58 | 5.46 |
| Shimai 001 | Yunnan Shifeng Seed Industry Co., Ltd. (YSSI) | 2012, 2013 | 5.43 | 7.13 |
| Shumai 1754 | YAU | 2018 | 7.19 | 4.78 |
| Wen 11chan55 | WAAS | 2016, 2017 | 5.94 | 4.63 |
| Wenmai 12 | WAAS | 2008, 2009 | 5.38 | 3.95 |
| Wenmai 13 | WAAS | 2012, 2013 | 2.55 | 7.62 |
| Yi 122-329 | ATECYm | 2014, 2015 | 6.51 | 4.75 |
| Yimai 1 | SMDY | 2006, 2007 | 6.1 | 4.46 |
| Yimai 2010-9 | SMDY | 2014, 2015 | 2.58 | 6.07 |
| Yimai 2013-34 | SMDY | 2016, 2017 | 5.69 | 4.33 |
| Yimai 2014-20 | SMDY | 2018 | 6.65 | 2.33 |
| Yimai 2016-9 | SMDY | 2018 | 7.39 | 2.21 |
| Yimai 6 | Yiliang Yifeng Seed Industry Co., Ltd. (YYSI) | 2012, 2013 | 5.35 | 7.43 |
| Yixi 2003-13 | SMDY | 2008, 2009 | 5.89 | 4.31 |
| Yixi 96-6 | SMDY | 2010, 2011 | 5.62 | 5.92 |
| Yixuan A03-2 | ATECYl | 2010 | 5.15 | 4.63 |
| Yu 058-8 | YuAAS | 2012, 2013 | 2.6 | 8.5 |
| Yu 09-5 | YuAAS | 2010, 2011 | 5.75 | 4.63 |
| Yu 15-2 | YuAAS | 2016 | 5.68 | 3.1 |
| Yu 17-2 | YuAAS | 2018 | 8.24 | 4.13 |
| Yu 17-4 | YuAAS | 2018 | 6.45 | 2.82 |
| Yumai 4 | YuAAS | 2014, 2015 | 6.2 | 3.82 |
| Yun 104-11 | IFC, YAAS | 2012, 2013 | 2.44 | 7.77 |
| Yun 104-15 | IFC, YAAS | 2012, 2013 | 2.57 | 8.82 |
| Yun 124-4 | IFC, YAAS | 2014, 2015 | 6.08 | 4.61 |
| Yun 124-6 | IFC, YAAS | 2014, 2015 | 5.63 | 3.92 |
| Yun 126-8 | IFC, YAAS | 2014, 2015 | 6.27 | 4.34 |
| Yun 154-15 | IFC, YAAS | 2016 | 5.82 | 4.13 |
| Yun 154-2 | IFC, YAAS | 2016 | 5.38 | 3.64 |
| Yun 154-64 | IFC, YAAS | 2016 | 5.67 | 3.15 |
| Yun 154-65 | IFC, YAAS | 2016, 2017 | 6.34 | 5.07 |
| Yunmai 101 | IFC, YAAS | 2008, 2009 | 5.45 | 3.63 |
| Yunmai 104 | Seed Management Department of Baoshan (SMDB) | 2014, 2015 | 5.66 | 3.35 |
| Yunmai 106 | SMDB | 2016, 2017 | 5.58 | 5.43 |
| Yunmai 108 | IFC, YAAS | 2018 | 7.76 | 3.01 |
| Yunmai 16I4-4 | IFC, YAAS | 2017 | 5.67 | 4.77 |
| Yunmai 16II4-1 | IFC, YAAS | 2017, 2018 | 6.3 | 4.48 |
| Yunmai 174I-11 | IFC, YAAS | 2018 | 7.15 | 3.41 |
| Yunmai 174I-12 | IFC, YAAS | 2018 | 6.05 | 2.81 |
| Yunmai 174I-2 | IFC, YAAS | 2018 | 6.29 | 1.75 |
| Yunmai 174I-7 | IFC, YAAS | 2018 | 6.39 | 4.73 |
| Yunmai 53 (CK) | IFC, YAAS | 2014, 2015, 2016 | 6.82 | 4.4 |
| Yunmai 56 (CK) | IFC, YAAS | 2017, 2018 | 7.02 | 5.96 |
| Yunmai 57 (CK) | IFC, YAAS | 2010, 2011, 2012, 2013 | 5.77 | 8.18 |
| Yunxuan 11-12 (CK) | IFC, YAAS | 2006, 2007, 2008, 2009 | 5.37 | 7.12 |
| Yunxuan 2 | IFC, YAAS | 2006, 2007 | 6.3 | 5.45 |
| Yunxuan 3 | IFC, YAAS | 2008, 2009 | 5.17 | 4.47 |
| Yunza 10 | IFC, YAAS | 2014, 2015 | 2.29 | 7.07 |
| Yunza 11 | IFC, YAAS | 2014, 2015 | 5.03 | 3.27 |
| Yunza 12 | IFC, YAAS | 2016, 2017 | 5.94 | 6.15 |
| Yunza 13 | IFC, YAAS | 2016 | 5.79 | 5.81 |
| Yunza 15 | IFC, YAAS | 2018 | 6.2 | 2.28 |
| Yunza 7 | IFC, YAAS | 2008, 2009 | 5.21 | 3.84 |
| Yunza 9 | IFC, YAAS | 2012, 2013 | 5.71 | 7.65 |
